# Perinatal exposure to traffic related air pollutants and the risk of infection in the first six months of life: a cohort study from a low-middle income country

**DOI:** 10.1007/s00420-024-02064-0

**Published:** 2024-04-17

**Authors:** Frida Soesanti, Gerard Hoek, Bert Brunekreef, Kees Meliefste, Jie Chen, Nikmah S. Idris, Nina D. Putri, Cuno S. P. M. Uiterwaal, Diederick E. Grobbee, Kerstin Klipstein-Grobusch

**Affiliations:** 1 Department of Child Health, Faculty of Medicine, Universitas Indonesia/Cipto Mangunkusumo General Hospital, Jakarta, Indonesia; 2 Julius Global Health, Julius Center for Health Sciences and Primary Care, University Medical Center Utrecht, Utrecht University, Utrecht, The Netherlands; 3 Environmental and Occupational Health Group Institute for Risk Assessment Sciences (IRAS), Utrecht University, Utrecht, The Netherlands; 4 Division of Epidemiology and Biostatistics, School of Public Health, Faculty of Health Sciences, University of the Witwatersrand, Johannesburg, South Africa; 5 Present Address: Department of Environmental Health, Harvard T. H. Chan School of Public Health, Boston, USA

**Keywords:** Low-middle income country, Traffic related air pollutants, PMs, Upper respiratory tract infection, Lower respiratory tract infection

## Abstract

**Objective:**

There is limited study from low-and-middle income countries on the effect of perinatal exposure to air pollution and the risk of infection in infant. We assessed the association between perinatal exposure to traffic related air pollution and the risk of infection in infant during their first six months of life.

**Methods:**

A prospective cohort study was performed in Jakarta, March 2016–September 2020 among 298 mother-infant pairs. PM_2.5_, soot, NO_x_, and NO_2_ concentrations were assessed using land use regression models (LUR) at individual level. Repeated interviewer-administered questionnaires were used to obtain data on infection at 1, 2, 4 and 6 months of age. The infections were categorized as upper respiratory tract (runny nose, cough, wheezing or shortness of breath), lower respiratory tract (pneumonia, bronchiolitis) or gastrointestinal tract infection. Logistic regression models adjusted for covariates were used to assess the association between perinatal exposure to air pollution and the risk of infection in the first six months of life.

**Results:**

The average concentrations of PM_2.5_ and NO_2_ were much higher than the WHO recommended levels. Upper respiratory tract infections (URTI) were much more common in the first six months of life than diagnosed lower respiratory tract or gastro-intestinal infections (35.6%, 3.5% and 5.8% respectively). Perinatal exposure to PM_2.5_ and soot suggested increase cumulative risk of upper respiratory tract infection (URTI) in the first 6 months of life per IQR increase with adjusted OR of 1.50 (95% CI 0.91; 2.47) and 1.14 (95% CI 0.79; 1.64), respectively. Soot was significantly associated with the risk of URTI at 4–6 months age interval (aOR of 1.45, 95%CI 1.02; 2.09). All air pollutants were also positively associated with lower respiratory tract infection, but all CIs include unity because of relatively small samples. Adjusted odds ratios for gastrointestinal infections were close to unity.

**Conclusion:**

Our study adds to the evidence that perinatal exposure to fine particles is associated with respiratory tract infection in infants in a low-middle income country.

**Supplementary Information:**

The online version contains supplementary material available at 10.1007/s00420-024-02064-0.

## Introduction

Air pollution related health problems have substantially contributed to the global burden of diseases over the past 25 years (Cohen et al. 2017; World Health Organization 2016, 2021; Chen and Hoek 2020). This increase is partially due to increasing air pollution in low-middle income countries (LMIC), like Indonesia (Cohen et al. 2017; World Health Organization 2016, 2021; Chen and Hoek 2020). Numerous epidemiological studies have shown that children exposed to tobacco smoke or higher levels of outdoor air pollution above recommended levels (e.g., by WHO) (Cohen et al. 2017; World Health Organization 2016, 2021) are more prone to develop respiratory disorders and infections in early life (Madsen et al. 2017; Nhung et al. 2017; Ratajczak et al. 2021; Soh et al. 2018; MacIntyre et al. 2014; Bowatte et al. 2015, 2018; Bai et al. 2018; Aguilera et al. 2013). Most studies to date investigating the effects of outdoor air pollution on respiratory health and ear infections during early childhood have focused mainly on postnatal exposure (Nhung et al. 2017; Bowatte et al. 2015, 2018; Bai et al. 2018; Yitshak-Sade et al. 2017). Exposure to postnatal PM_10_, PM_2.5_, and NO_2_ has been associated with an increased risk of bronchiolitis (Yitshak-Sade et al. 2017). Furthermore, postnatal exposure to PM_2.5_ (Shi et al. 2021) and NO_2_ is linked to a higher risk of otitis media (Bowatte et al. 2018; Shi et al. 2021). A study in China revealed that PM_2.5_ exposure was associated with a higher incidence of childhood pneumonia (Shi et al. 2021), while another study showed that exposure to PM_10_ and SO_2_ during the first year of life was associated with an increased risk of pneumonia in children aged 3–6 years (Liu et al. 2022). Similarly, a study in Brazil demonstrated that exposure to PM_10_ and SO_2_ in the first year of life is linked to an increased risk of pneumonia (Souza and Nascimento 2016).

There is growing evidence on the association between exposure to air pollution during pregnancy and the incidence of respiratory tract infections in their off springs during early life (Madsen et al. 2017; Soh et al. 2018; Aguilera et al. 2013; Esplugues et al. 2011; Goshen et al. 2020; Lu et al. 2021). The period of in utero and early post natal is critical in the development of organs system, including respiratory and immune systems (Esplugues et al. 2011; Lu et al. 2021). Therefore, potential harmful effects of toxic pollutions during pregnancy might result in long-lasting impaired capacity to fight infections and increased risk of allergic manifestations later in life (Lu et al. 2021; Delfino et al. 2011).

Important evidence on the role of exposure to traffic related air pollution during the intrauterine period with early life infection comes from the ESCAPE study that consists of 10 European birth cohorts (MacIntyre et al. 2014). This study showed that every 10 μg/m^3^ increase in NO_2_ is consistently associated with higher risk of pneumonia and otitis media [OR of 1.30 (95% CI: 1.03; 1.65) and 1.09 (95% CI: 1.02; 1.16), respectively] (MacIntyre et al. 2014). Another study showed an association of PM_2.5_ with bronchitis (Soh et al. 2018), lower respiratory tract infection (LRTI) (Liu et al. 2022), and ear infection in the first years of life (Soh et al. 2018; Aguilera et al. 2013). Other studies, however, found no significant association between intrauterine exposure to traffic related air pollution with the incidence of LRTI, bronchiolitis, bronchitis, wheezing and persistent cough (Madsen et al. 2017; Esplugues et al. 2011). Morales et al. (Morales et al. 2015) showed that intrauterine exposure to NO_2_ was associated with increased risk of low lung function (OR of 1.30 (95%CI: 0.47; 1.76)) in preschool age children, while early postnatal exposure to NO_2_ was not significantly associated with low lung function.

Studies of traffic related air pollution effects in infant health were mainly conducted in high income countries (HIC) with limited evidence from middle income countries, especially lower middle income countries (LMIC) where the burden of infectious diseases is high and air pollution levels have massively increased (Cohen et al. 2017; World Health Organization 2016, 2021). Most research from middle income countries originates from China, which is an upper-middle income country (UMIC). A study by Liu et al. (2020) showed that higher levels of NO_2_ during various trimesters of gestation and the first year of life increased the risks pneumonia in childhood. Other studies have also reported that prenatal exposure to PM_2.5_ was linked to higher risk of childhood pneumonia and ear infection (Lu et al. 2023a, b). The growing evidence on the role of outdoor air pollution in the incidence of infection in childhood as well as its severity warrants investigation on the magnitude of its effect in LMIC. The sources of air pollution in LMIC include motor vehicle exhaust, emitting i.e. NO_2_, PM_2.5_ and soot (Soh et al. 2018; Soesanti et al. 2023). Other than traditional prevention measures such as vaccination, hygiene, sanitation, and nutrition; improving the air quality through public environmental policies probably has an important role to reduce high mortality rates under 5 years together with traditional prevention measures. Therefore, we performed a population-based cohort study in Indonesia to investigate the association between perinatal exposure to traffic related air pollution with the incidence of infections in infants during their first six months of life.

## Material and methods

We performed a population-based cohort study in nine primary healthcare centers (Cempaka Putih, Johar Baru, Kemayoran, Kramat, Jatinegara, Kampung Melayu, Matraman, Paseban, Rawa Bunga) in Jakarta, Indonesia, from March 2016 until September 2020 (Soesanti et al. 2023). Jakarta, the capital city of Indonesia, stands out as a prime example of significant exposure to air pollution, with around 70–80% of the overall air pollution in Jakarta to be attributed to traffic sources. This is driven by a large number of vehicles, including 16.1 million motorcycles, 4.3 million cars, and public transportation systems that contribute substantially to pollution levels in the broader Jakarta region (Soesanti et al. 2023; Central Bureau of Statistics 2003).

We obtained ethical approval from the Institutional Review Board of the Faculty of Medicine University of Indonesia/Cipto Mangunkusumo General Hospital, Jakarta, Indonesia (reference number: 895/UN2.F1/ETIK/2015). Written informed consent was obtained from all the participants before their enrolment (Soesanti et al. 2023).

This study began by enrolling 413 pregnant women who lived within the primary care center catchment area and were easily reachable by phone (Soesanti et al. 2023). Recruitment was performed by midwives at the early stages of pregnancy (in the first or early second trimester with gestational age < 20 weeks). Over the years, the number of recruited pregnant women varied, with 69 participants in 2016, 132 in 2017, 122 in 2019, and 90 in 2019 (Soesanti et al. 2023). Forty-nine participants dropped out for various reasons (refused to continue or moved out of town), 13 were excluded because of early miscarriages, and one was excluded because of an ectopic pregnancy, resulting in a final sample size of 348 mother-singleton infant pairs. Further exclusions were made due to missing data on air pollution measurements (4 pairs), incomplete data on outcome measurement (28 pairs), and extremely low birth weight (1 pair, BW 700 g, GA 27 weeks). As a result, 315 mother-infant pairs were included in this current study. The pregnant women were followed until delivery, and their infants were followed up until six months of age.

### Assessment of demographic and pregnancy-related information

A full description of the demographic, socio-economic, and pregnancy-related information has been described in detail previously published paper (Soesanti et al. 2023). We used structured questionnaires during enrollment to collect data on various maternal characteristics, including maternal age, parity, abortion, working status, household income, level of education, prior drug use, and smoking (active and passive) history prior to pregnancy (Soesanti et al. 2023). Paternal characteristics, including age, level of education, and smoking habits prior to their spouse’s pregnancy were also obtained at enrolment (Soesanti et al. 2023). Level of education was categorized as elementary, high school, and under-post graduate, while family income was categorized as below or above the minimum monthly wedges per capita in Jakarta (≥ 290 USD) (Soesanti et al. 2023).

We used a structured questionnaire administered at each trimester to assess pregnancy-related factors, including pregnancy complications, medications, and smoking habits (Soesanti et al. 2023). Weight gain during pregnancy, denoted as delta BMI was derived by subtracting the pre-pregnancy BMI from the BMI recorded at delivery. Most pregnant women delivered at the primary care center. Referral to a secondary health care center for complicated pregnancy or delivery was following the national health care policy (Soesanti et al. 2023). At delivery, maternal blood pressure, body temperature, gestational age, and complications during delivery were documented (Soesanti et al. 2023).

### Assessment of infection outcomes in infants

We used repeated interviewer-led questionnaires to obtain evidence of infection at each follow-up, performed at 1 month (infection between 0 and 1 month of age), 2 months (between 1 and 2 months of age), 4 months (between 2 and 4 months of age) and 6 months (between 4 and 6 months of age) in accordance with the national program on vaccination. We obtained information on infection as categorical “yes” and “no”. We asked questions about history of fever (defined as axilla temperature > 38 °C), symptoms of cough, runny nose, wheezing, shortness of breath, diarrhea, and vomiting during the time interval. The symptoms were categorized into respiratory tract infection (upper and lower), gastrointestinal infection, and other infections.

Lower respiratory tract infection (LRTI) was defined as pneumonia or bronchiolitis diagnosed by a physician. To obtain this information, we asked mothers two specific questions: (1) Has your infant been diagnosed with pneumonia by a physician? (2) Has your infant been diagnosed with bronchiolitis by a physician? Additionally, we cross-checked this data with hospital medical records. We also documented the medication received and the duration of hospitalization associated with pneumonia or bronchiolitis. For upper respiratory tract infection (URTI) symptoms, we asked about fever, cough, runny nose, and stuffed nose by asking mothers the following questions: (1) Has your infant had a fever? If the response was “yes”, we proceeded with the following questions: (1) Has your infant had a cough? (2) Has your infant had a runny nose? (3) Has your infant had a stuffed nose? Gastrointestinal (GI) infection was defined based on the presence of specific symptoms obtained through the following questions: (1) Has your infant had diarrhea? (2) Has your infant had vomiting?

We also recorded any episodes of physician-diagnosed urinary tract infection, sepsis, central nervous system (CNS) infections, tuberculosis, and dengue fever. We categorized the infection episodes as other non-specific infections if the infants only have a fever without any other symptoms that can be classified as URTI, LRTI, or GI infection or without any specific diagnosis from a physician. Any episodes of hospitalization, length of stay, diagnosis, and medication given during hospitalization were also recorded.

### Traffic-related air pollution assessment

**Fig. 1:**
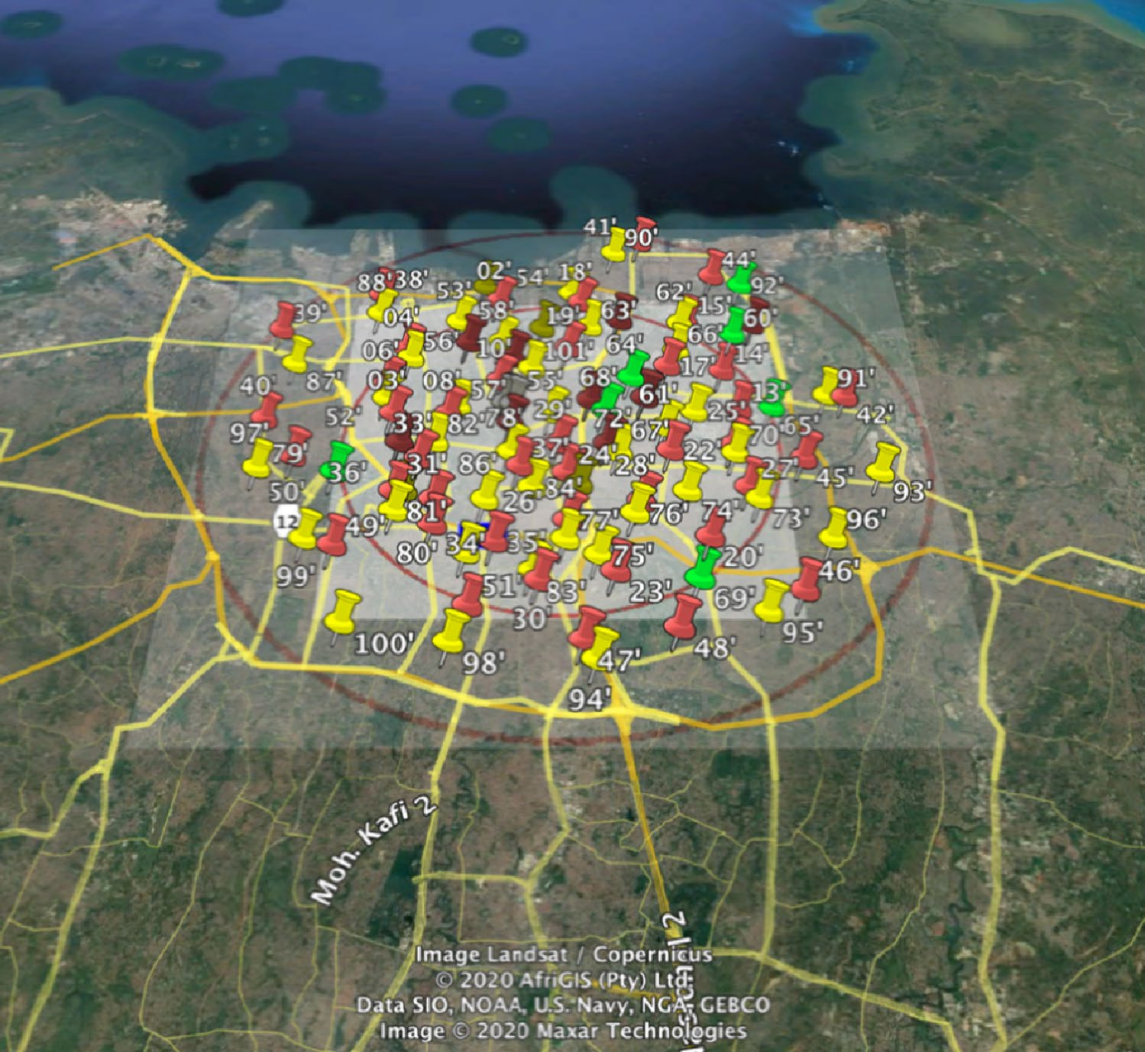
Study area of Jakarta air pollution sampling (Soesanti et al. 2023). Red pin indicates traffic sites, yellow pin: urban background, green pin: urban green, white pin indicates reference site (colour figure online)

The cohort’s exposure to air pollution was assessed using land use regression (LUR) models. These models were developed based on targeted measurements of fine particles and nitrogen oxides in Jakarta (Lu et al. 2023b). Detailed description of the measurements and model development have been described previously in Soesanti et al. (2023) Briefly, the study area encompassed the primary care catchment area of the cohort study, located in the center of Jakarta. Measurements were made at 88 sites across the study area (Fig. 1) and LUR models were developed for PM_2.5_, soot (a measure of black carbon), nitrogen dioxide (NO_2_) and the sum of nitrogen dioxide (NO_2_) and nitrogen oxide (NO), denoted as NO_x_.

LUR models were developed using supervised linear regression procedures, using predictor variables derived from direct field observations of traffic counts and street configuration and global GIS databases of road data from Open Street map and impervious surface information (Soesanti et al. 2023). These models can be found in Table S1. All the models included motorcycle counts at the nearest road as a predictor variable. The LUR models explained 61, 59, 26 and 33% of the measured annual average concentration variability for NO_x_, NO_2,_ PM_2.5_ and soot, respectively (Soesanti et al. 2023). Exposure to air pollution was assessed individually using LUR model. However, due to the lack of continuous monitoring data for all four evaluated pollutants in Jakarta, we could not use extrapolation methods and were not able to account for temporal differences between women giving birth at different times (Soesanti et al. 2023). As a result, we could not calculate trimester-specific or full pregnancy-specific exposure. We also could not define specifically the exposure in the first six months of postnatal life. Continuous monitoring data was available from the US embassy only for PM_2.5_ (AirNow Department of State 2023). We calculated annual average concentration and full pregnancy average concentrations related to temporal variation (Soesanti et al. 2023).

### Confounders

We categorized the confounding variables into three groups: (1) family demographic factors, (2) environmental factors, and (3) infant's factors. Within family demographic factors, variables such as level of education, household income, maternal age at pregnancy, parity (Madsen et al. 2017; MacIntyre et al. 2014; Aguilera et al. 2013; Shi et al. 2021; Lu et al. 2023a), delta BMI, mother's working status during pregnancy, and maternal co-morbidity/gestational complications were predetermined as potential confounders (Madsen et al. 2017; MacIntyre et al. 2014; Aguilera et al. 2013; Shi et al. 2021). Level of education and household income were used as proxies for socioeconomic status (Madsen et al. 2017; MacIntyre et al. 2014; Shi et al. 2021). Smoking status during pregnancy (active or passive) (Madsen et al. 2017; MacIntyre et al. 2014; Shi et al. 2021; Liu et al. 2022) and exposure to pesticides during pregnancy, as reported in the questionnaire, were considered potential confounders related to indoor environmental factors. Gestational age and birth weight were identified as potential confounders related to infant factors and were treated as numerical variables (Madsen et al. 2017; MacIntyre et al. 2014; Aguilera et al. 2013; Liu et al. 2022).

### Statistical analysis

Continuous variables for baseline subject characteristics of the mothers and infants were expressed as mean and standard deviation or median and interquartile range if distributions were skewed, while categorical variables were expressed as number of subjects and its percentage. Air pollutant concentrations were presented as annual average concentrations of PM_2.5_, soot, NO_x_, and NO_2_ with interquartile range (IQR) and minimum–maximum values. The distribution of infection during the first six months of life was tabulated for each age (0–1,1–2, 2–4, and 4–6 months) interval, presented as numbers and percentages.

We included infants with a birth weight ≥ 2500 g for the final analysis, as children with low birth weight have a higher risk of infections in infancy. This restriction aimed to minimize the potential for bias. We analyzed the cumulative incidence of upper respiratory tract, lower respiratory tract, and gastrointestinal infection in the first six months of life separately. The cumulative incidence of infection was denoted as binomial “yes” and “no” for each infection, while air pollutant concentration was entered as a continuous variable.

Multivariable logistic regression adjusted to all potential confounders was performed to investigate the association between traffic-related air pollutants with the cumulative incidence of URTI, LRTI, and GI infections during the first six months of life. Additionally, we analyzed the association between traffic-related air pollutants with the incidence of URTI at each age interval (1–2 months, 2–4 months, 4–6 months) by performing multivariable logistic regression adjusted to all potential confounders. Effect estimates (Odds ratios) were calculated based on the IQR increment of each air pollutant (Soesanti et al. 2023). The IQR was 7.14 μg/m^3^ for PM_2.5_, 0.75 × 10^–5^ per m for soot, 4.68 μg/m^3^ for NO_x_, and 3.74 μg/m^3^ for NO_2_ (Lu et al. 2023b). Statistical significance was determined when 95% confidence intervals did not include unity, indicated two-sided *p* values less than 0.05. We used R.4.1.2. to develop LUR models and IBM SPSS version 24 for Mac for statistical analyses.

## Results

There were 315 mother-infant pairs included at initial, but for final analysis, we restricted our analysis to those with BW of ≥ 2500 g, resulting in 298 pairs of mother-infant for final analysis. The average age of pregnant women in our study was 26.7 (SD 6.6), and they were mostly high school graduates and were on their first pregnancy without any history of abortion (Table 1). Around 64% reported a low family income and 41.5% of women worked during their pregnancy. Five (2.1%) women had chorioamnionitis and 6% suffered from gestational hypertension. Infants were born with an average weight of 3219.1 g (SD 456.9) with an average gestational age of 38.6 (1.5) weeks. Seventeen of the infants were born with low birth weight. Only 5 women (1.6%) actively smoked during pregnancy while 62.2% of fathers smoked during their wife’s pregnancy. There were 45.1% of subjects who routinely used pesticides during pregnancy.

**Table 1:** Baseline subject characteristics

Variable	Total (n = 315)
*Mother characteristics*	
Mother’s age (years)	26.7 (6.6)
Mother’s enrolment period, n (%)	
Trimester 1	153 (48.6)
Trimester 2	162 (51.4)
Parity, n (%)	
0–1	215 (68.5)
≥ 2	100 (31.5)
No history of abortion, n (%)	253 (80.6)
Mother’s education level, n (%)	
Elementary	20 (6.3)
High school	226 (71.7)
Under-post graduate	69 (21.9)
Family income (USD/month), n (%)	112 (36.0)
> 290 USD	
Working during pregnancy, n (%)	131 (41.5)
BMI at pre-pregnancy (kg/m^2^)	22.9 (4.5)
Delta BMI (kg/m^2^)	4.81 (2.2)
Pregnancy complications, n (%)	
Hypertension	19 (6.0)
PROM	41 (13.0)
Oligohydramnios	19 (14.6)
Chorioamnionitis	5 (2.1)
*Infant characteristics*	
Male sex, n (%)	159 (50.5)
Gestational age based on FDLM (weeks)	38.6 (1.5)
Birth weight (grams)	3219.1 (456.9)
Birth length (cm)	49.0 (1.8)
Low birth weight (BW < 2500 g), n (%)	17 (10.9)
Premature (GA < 37 weeks), n (%)	12 (7.8)
Smoking habits	
Mother smoking during pregnancy, n (%)	5 (1.6)
Father smoking during pregnancy, n (%)	196 (62.2)
Number of other household members smoking during pregnancy, n (%)	
1 member	145 (46.0)
≥ 2 members	79 (25.2)
Total cigarettes/day	10.8 (9.39)
Other environmental factors	
Home fuel resources, n (%)	
Gas	259 (82.2)
Kerosene	7 (2.2)
Household insecticides usage, n (%)	142 (45.1)
Routine garbage burning, n (%)	7 (2.2)

Numbers are expressed in mean (SD) otherwise indicated

*PROM* premature rupture of the membrane, *FDLM* first day of last menstruation, *GA* gestational age; *Total cigarettes/day* average total cigarettes consumed by household member per day

The average concentration of PM_2.5_ and NO_2_ in our study was well above the recommended WHO air quality guideline level (World Health Organization 2021), as we have reported previously (Lu et al. 2021). The mean of PM_2.5_ concentration was around seven times (36.45 µg/m^3^) higher than the recommended level by WHO guidelines of 5 µg/m^3^ annually (World Health Organization 2021), while the mean NO_2_ concentration in our study was at least three times (37.27 µg/m^3^) higher than the recommended level by WHO guidelines (10 µg/m^3^ annually) (World Health Organization 2021) (Table 2). The IQR of each air pollutants are also shown in Table 2. The standard deviation is about 10% of the mean for all air pollutants (Table 2). Soot was moderately correlated (r = 0.51) with PM_2.5_, and NO_x_ was highly correlated (r = 0.87) with NO_2,_ while the correlation between the rest of the pollutants was weak (S2 & S3 Tables) (Soesanti et al. 2023).

**Table 2:** Distribution of the air pollutant concentrations

Air pollutant	Concentration of air pollutants (n = 315)		
	Mean ± SD	Min–max	Interquartile range (IQR)
PM_2.5_ (μg/m^3^)	36.45 ± 3.72	32.45–51.28	7.14
Soot (10^–5^ per m)	4.57 ± 0.53	3.74–6.37	0.74
NOx (μg/m^3^)	37.27 ± 4.21	23.25–54.62	4.47
NO_2_ (μg/m^3^)	32.71 ± 3.28	21.30–44.84	3.65

Table 3 shows that the incidence of infection was increased at each age interval (9.2% vs 21.6% vs 43.5% vs 51.7% at 1, 2, 4, 6 months of age interval, respectively), partly because of the four- and six-months referred to two months interval instead of one month interval. The rates of hospitalization and antibiotics used also increased with the rise in infection cases. Upper respiratory tract infection (URTI) was the most common infection during the first six months of life (35.6% at 4–6 months of age), followed by gastrointestinal infection (5.8% at 4–6 months of age), and lower respiratory tract infection (3.5% at 4–6 months of age). There was one death at four months due to diarrhea with severe dehydration and one death at six months due to dengue shock syndrome. Both deaths occurred as a result of a delay reaching out to health care facility.

**Table 3:** Distribution of the incidence of infection in infant during their first six months of life

Variables	0–1 month (n = 315)	1–2 months (n = 315)	2–4 months (n = 315)	4–6 months (n = 315)
Episodes of infections, n (%)	29 (9.2)	68 (21.6)	137 (43.5)	163 (51.7)
URTI, n (%)	9 (2.9)	56 (17.7)	119 (37.8)	112 (35.6)
LRTI, n (%)	1 (0.3)	2 (0.6)	4 (1.3)	11 (3.5)
GI infection, n (%)	4 (1.3)	5 (1.6)	9 (2.9)	18 (5.8)
UTI, n (%)	1 (0.3)	0 (0)	1 (0.3)	0 (0)
Sepsis, n (%)	8 (2.5)	0 (0)	0 (0)	0 (0)
Other non-specific infections, n (%)	6 (1.9)	3 (0.9)	4 (1.3)	22 (6.9)
Episodes of hospitalization, n (%)	33 (10.5)	3 (0.9)	6 (1.9)	12 (3.8)
Reason for hospitalization, n (%)				
Hyperbilirubinemia	22 (7.0)	0	0	0
LRTI	1 (0.3)	0	4 (1.3)	7 (2.2)
Sepsis	8 (2.5)	0	0	0
UTI	1 (0.3)	0	0	0
Congenital anomaly	1 (0.3)	0	1 (0.3)	0
URTI	0	1 (0.3)	0	2 (0.6)
Tuberculosis	0	1 (0.3)	0	2 (0.6)
GI infection	0	1 (0.3)	0	1 (0.3)
Dengue fever	0	0	1(0.3)	1 (0.3)
Antibiotic usage, n (%)	10 (3.2)	13 (4.1)	29 (9.1)	40 (12.7)
Infant death, n (%)	0 (0)	0 (0)	1 (0.3)	1 (0.3)
Causes of death			Diarrhea, severe dehydration	Dengue shock syndrome

*URTI* upper respiratory tract infection; *LRTI* lower respiratory tract infection; *GI* gastrointestinal infection; *UTI* urinary tract infection

Table 4 shows the association between air pollutants concentrations with cumulative risk of specific infections, i.e., URTI, LRTI, or gastrointestinal infection in the first six months of life. PM_2.5_ was positively associated with increased risk of URTI and LRTI with adjusted OR of 1.50 (0.91; 247) and 1.14 (0.79; 1.64) for every IQR increase, respectively. We did not observe the same result for gastrointestinal infection. However, all confidence intervals included unity. The risk of having LRTI was increased with every IQR increased of PM_2.5_, soot, NO_x,_ and NO_2_ (aOR of 1.45, 1.46, 1.43, 1.52, respectively), but confidence intervals were wide and included unity. We also assessed the association between all air pollutants with the incidence of URTI at each age intervals (Table 5). Perinatal exposure to soot was significantly associated with increased risk of URTI at 4–6 months age interval with aOR of 1.45 (95% CI of 1.02; 2.09) for every IQR increment. We excluded the 0–1 month age interval from this analysis because of very small number of infants with URTI (as shown in Table 3) to avoid over adjustment. Additionally, we looked at all infection combined in the first six months of life and found no material association between all air pollutants and the cumulative incidence of all infection combined (S4 Table).

**Table 4:** The association between perinatal exposure to air pollutants with URTI, LRTI and gastrointestinal infection during the first sixth months of life

Air pollutant concentration	Odd ratio (95% confidence interval)		
	Upper respiratory tract infection (n = 185)	Lower respiratory tract infection (n = 14)	Gastrointestinal infection (n = 26)
*PM*_*2.5*_			
Crude	1.45 (0.92; 2.28)	1.39 (0.54; 3.56)	0.84 (0.38; 1.87)
Adjusted	1.50 (0.91; 2.47)	1.45 (0.53; 4.00)	0.97 (0.42; 2.23)
*Soot*			
Crude	1.17 (0.84; 1.63)	1.34 (0.66; 2.73)	0.99 (0.56; 1.72)
Adjusted	1.14 (0.79; 1.64)	1.46 (0.69; 3.10)	1.08 (0.57; 2.02)
*NO*_*x*_			
Crude	0.83 (0.64; 1.07)	1.40 (0.78; 2.51)	1.15 (0.75; 1.77)
Adjusted	0.80 (0.60; 1.06)	1.43 (0.75; 2.72)	1.20 (0.73; 1.98)
*NO*_*2*_			
Crude	0.85 (0.65; 1.10)	1.48 (0.78; 2.76)	1.17 (0.74; 1.84)
Adjusted	0.81 (0.61; 1.09)	1.52 (0.77; 3.02)	1.25 (0.74; 2.10)

All effect estimates of continuous data correspond to interquartile range (IQR) increase i.e. 7.14 μg/m^3^ for PM_2.5_, 0.74 × 10^–5^ per m for PM_2.5abs_, 4.47 μg/m^3^ for NO_x_, and 3.65 μg/m^3^ for NO_2_

Adjusted to mother’s age at pregnancy, household SES, mother working status during pregnancy, parity, delta BMI, active smoking, passive smoking, exposure to insecticides during pregnancy, gestational age, delivery complications, birth weight

**Table 5:** The association between perinatal exposure to air pollutants with upper respiratory tract infection at each age interval during the first sixth months of life

Air pollutants concentration	Odd ratio (95% confidence interval)		
	1–2 month of age (n = 56)	2–4 month of age (n = 119)	4–6 month of age (n = 112)
*PM*_*2.5*_			
Crude	1.36 (0.87; 2.11)	0.84 (0.91; 2.22)	1.23 (0.78; 1.92)
Adjusted	1.33 (0.83; 2.13)	1.36 (0.84; 2.19)	1.31 (0.80; 2.13)
*Soot*			
Crude	1.07 (0.77; 1.48)	1.19 (0.86; 1.66)	1.35 (0.96; 1.88)
Adjusted	1.00 (0.70; 1.42)	1.13 (0.79; 1.60)	1.45 (1.02; 2.09)*
*NO*_*x*_			
Crude	1.00 (0.78; 1.29)	1.01 (0.79; 1.31)	0.87 (0.67; 1.12)
Adjusted	1.03 (0.78; 1.34)	1.02 (0.77; 1.34)	0.83 (0.63; 1.11)
*NO*_*2*_			
Crude	1.02 (0.79; 1.33)	1.05 (0.80; 1.36)	0.88 (0.67; 1.15)
Adjusted	1.04 (0.79; 1.38)	1.05 (0.79; 1.39)	0.85 (0.63; 1.14)

All effect estimates of continuous data correspond to interquartile range (IQR) increase i.e. 7.14 μg/m^3^ for PM_2.5_, 0.74 × 10^–5^ per m for PM_2.5abs_, 4.47 μg/m^3^ for NO_x_, and 3.65 μg/m^3^ for NO_2_

Adjusted to mother’s age at pregnancy, household SES, mother working status during pregnancy, parity, delta BMI, active smoking, passive smoking, exposure to insecticides during pregnancy, gestational age, delivery complications, birth weight

*p value < 0.05

## Discussion

This study provides suggestive evidence that perinatal exposure to PM_2.5_ and soot are associated with the incidence of respiratory tract infections in the first six months of life, especially the upper respiratory tract infections (URTI), with the association between soot and URTI was substantially significant at 4–6 months age interval. To our knowledge, our study is the first study to evaluate the effect of exposure to multiple important traffic related air pollutants during the perinatal period on the incidence of infection in the first six months of life in a low-middle income country setting. Most of epidemiological evidence comes from high income countries (Madsen et al. 2017; MacIntyre et al. 2014; Aguilera et al. 2013; Esplugues et al. 2011; Goshen et al. 2020) and many of them focus more on the postnatal exposure (Nhung et al. 2017; Ratajczak et al. 2021; Bowatte et al. 2015, 2018; Bai et al. 2018) instead of prenatal exposure or the perinatal period of exposure. However, there is a growing body of research, particularly from countries like China (an upper-middle income country) that assesses prenatal, perinatal, and postnatal exposure to air pollutants, (Shi et al. 2021; Liu et al. 2020, 2022; Lu et al. 2021, 2023a, b; Soesanti et al. 2023) but the evidence from low-middle income countries remains relatively limited.

Our prospective cohort study design commencing in early pregnancy is a major strength of our study (Soesanti et al. 2023), which is very useful for studying the exposure–response relationship in disease development. This design allowed us to collect individual data on the incidence of infection at 0–1 month, 1–2 month, 2–4 month, and 4–6 month of age intervals. The measurements at multiple time points allowed us to evaluate the consistency of associations. We also consider the direct measurement of multiple important (traffic related) air pollution concentrations (PM_2.5_, soot, NO_x_, NO_2_) using standardized methodology as one of our strengths (Soesanti et al. 2023; Aguilera et al. 2009; Ballester et al. 2010). Numerous epidemiological studies rely on historical data for air pollutant concentration and typically focus only on one or two specific air pollutants (Aguilera et al. 2009; Ballester et al. 2010). In our study, we used LUR modeling to assess individual levels of exposure. We based our estimate on the residential address of the pregnant women to minimize the possibility of exposure misclassification (Soesanti et al. 2023; Aguilera et al. 2009; Ballester et al. 2010).

There are a few limitations in our study. Firstly, while our cohort size is significant within the Indonesian context, the relatively small sample size resulted in less precise estimates, as evidenced by the wide confidence intervals (Soesanti et al. 2023). Secondly, instead of using clinical records, we relied on questionnaires to collect individual data on health outcomes. The infection data, except for LRTI or other infections that lead to hospitalization was collected based on the symptoms of upper respiratory tract infection, gastrointestinal infection, or other specific infections. This can result in potential misclassification and recall bias (Aguilera et al. 2013). We minimized the possibility of misclassification by using repeated standardized questionnaires for infections performed by trained research personnel who are unaware of the air pollutants exposure level of each subject. However, we cannot confidently exclude all the possibility of residual bias. Furthermore, our study verified data on LRTI not only through questionnaires but also by cross-referencing with hospital medical records for increased data accuracy. Thirdly, we did not measure the postnatal concentration of the air pollution. Although a study by Esplugues et al. (2011) indicates that air pollution exposure during the first year of life was highly correlated with prenatal exposure, but we could not confidently specify whether the increased risk of infection at 6 months of age was associated with prenatal exposure only or the continuum exposure since prenatal to early postnatal period. Thus, we defined the exposure as a perinatal exposure.

Our study showed that perinatal exposure to fine particles, specifically soot (black carbon), was associated with an increased risk of developing URTI in infants for their first six months of life. This result adds to the evidence of the detrimental effect of perinatal exposure to (traffic related) air pollution in infants on early life infection, especially on the respiratory tract infection. Most of the previous studies focused more on the incidence of lower respiratory tract, such as pneumonia, bronchiolitis, and hospitalization associated with pneumonia, instead of URTI. A study in Singapore reported a significant association of prenatal PM_2.5_ with ear infection and bronchiolitis/bronchitis (Soh et al. 2018). Similarly, a study in China reported a link between PM_2.5_ and increased risk of ear infections (Lu et al. 2023b). On the contrary, the findings from the ESCAPE study showed no significant association between PM_s_ with diseases categorized as URTI and Pneumonia (MacIntyre et al. 2014). Goshen et al. (2020) showed that intrauterine exposure to PM_2.5_ is adversely associated with the incidence of LRTI in a Arab-Bedouin population, while a cohort study from Jedrychowsky et al. (Jedrychowski et al. 2013) reported that intrauterine exposure to PMs is associated with increased risk of pneumonia at 7 years of age. A study by Lu et al. (2023a) demonstrated that exposure to PM_2.5_ during prenatal and postnatal periods (defined as the first year of life) is linked to an elevated risk of pneumonia at aged 3–6 years old. Specifically, the adjusted odds ratio (95% CI) for pneumonia was 1.17 (1.04; 1.30) for each interquartile range (IQR) increase in prenatal PM _2.5_ exposure, and 1.12 (1.02; 1.22) for each IQR rise in postnatal PM_2.5_ exposure (Lu et al. 2023a). Our study showed no meaningful relationship between perinatal exposure to PM_2.5_ and soot with the risk of LRTI. However, our results indicated a positive association.

The biological mechanism of prenatal and early postnatal exposure to air pollution with early life infection is still not well understood. Chronic exposure to external factors during pregnancy such as PM_s_ and NO_2_ may disrupt biological mechanisms that regulate fetal growth, maturation and development (Hertz-Picciotto et al. 2005; Ashley-Martin et al. 2016; Friedman et al. 2021; Korten et al. 2017; Latzin et al. 2011; Lee et al. 2011). Specifically, exposure to PM_s,_ including soot, during pregnancy, may induce oxidative stress that causes inefficient repair mechanisms of the developing lung and genetic modification that probably contributes to increased pulmonary susceptibility (Hertz-Picciotto et al. 2005; Ashley-Martin et al. 2016; Friedman et al. 2021; Korten et al. 2017; Latzin et al. 2011; Lee et al. 2011). In addition, environmental stimuli may induce acquired epigenetics states that affect gene expression and phenotypic outcome (Korten et al. 2017). PM_2.5_ have been known to cause systemic inflammation that produce free oxygen radicals that induce oxidative stress and then produce inflammation (Hertz-Picciotto et al. 2005; Ashley-Martin et al. 2016; Friedman et al. 2021; Korten et al. 2017; Latzin et al. 2011; Lee et al. 2011).

Several studies from Germany (Latzin et al. 2011), Czech Republic (Hertz-Picciotto et al. 2005), Canada (Ashley-Martin et al. 2016), and US (Lee et al. 2011), have reported that exposure to air pollution during pregnancy was associated with increased levels of immune biomarkers in the mother and the neonate. Friedman et al. (2021) reported that the outdoor concentration of PM_2.5_ in early pregnancy is associated with maternal levels of inflammatory cytokines, during mid-pregnancy, but less consistent findings were reported with inflammatory biomarkers in cord blood at delivery (Hertz-Picciotto et al. 2005; Ashley-Martin et al. 2016; Friedman et al. 2021; Korten et al. 2017; Latzin et al. 2011; Lee et al. 2011). Mice that were exposed to urban PM_s_ showed elevated cytokine levels and increased levels of lipid and protein oxidation. The ENVIRONAGE birth cohort study (Bové et al. 2019) showed that soot particles were detected on the maternal and fetal sides of the placenta, suggesting that soot may be transported to the developing fetus. PM_2.5_ can cross the placenta and enter the bronchi and alveoli of the fetus compared to larger PM_s_ and have been associated with abnormal lung genesis and hyperactive lung disease (Hertz-Picciotto et al. 2005; Ashley-Martin et al. 2016; Friedman et al. 2021; Korten et al. 2017; Latzin et al. 2011; Lee et al. 2011).

Our study did not find statistically significant associations of perinatal exposure to traffic related NOx and NO_2_ with the incidence of URTI, LRTI, or gastrointestinal infection during the first six months of life. Similar results were reported by the MoBa study (Madsen et al. 2017) in Norway. The ESCAPE study (MacIntyre et al. 2014), however, reported an elevated and statistically significant association of NO_2_ with pneumonia (OR = 1.30, 95% CI: 1.02; 1.65 per 10 µg/m^3^ increase in NO_2_) as well as with otitis media (OR = 1.09, 95% CI of 1.02; 1.16 per 10 µg/m^3^). The findings of the ESCAPE study are consistent with the results of other studies in HIC settings (Bowatte et al. 2018; Aguilera et al. 2013; Brauer et al. 2008). Additionally, exposure to higher levels of NO_2_ during prenatal and early postnatal period was significantly associated with an increased risk of pneumonia in childhood (Liu et al. 2020).

The biological mechanisms linking prenatal/perinatal exposure to air pollution with gastrointestinal tract infections are not well understood and have been less studied compared to the association with respiratory tract inflammation. Only very few studies assessed the relation of perinatal exposure to air pollution with gastrointestinal infections and most of the studies focused on the development of inflammatory bowel disease (Fouladi et al. 2020; Mutlu et al. 2018, 2011). Exposure to PM_s_ increased the production of mitochondrial reactive oxygen species (ROS) and the release of inflammatory cytokines increasing overall gut permeability which in turn affects the fetal organ development including the gastrointestinal system (Mutlu et al. 2018, 2011). This hypothesis aligns with the Barker hypothesis of Developmental Origins of Health and Disease (DOHaD) in adult (Fouladi et al. 2020; Mutlu et al. 2018, 2011).

In summary, children from low-and-middle income countries and in poor resource settings might carry an increased risk of air pollution-related effects, not only because they are exposed to higher exposure to air pollution but also because of additional risk factors for infection such as exposure to smoke (active or passive) and poor diets due to a low socioeconomic background. Our study provides additional evidence that perinatal exposure to fine particles, especially soot (black carbon), as early as in pregnancy and early six months of postnatal life might increase the risk of upper and lower respiratory tract infection. This evidence warrants health professionals and policy makers to raise awareness on the hazardous effects of air pollutions for the growing fetus and subsequent child health. Further research on a larger scale is needed to fully understand how exposure to air pollution during crucial developmental periods, such as prenatal and early postnatal life, can affect the risk of specific infections in childhood. Continuing to monitor and study the groups from our research longitudinally is crucial for understanding the long-term effects of early-life exposure to air pollutants on childhood infections.

### Supplementary Information

Below is the link to the electronic supplementary material.

Supplementary file1 (DOCX 23 KB)

## Data Availability

The datasets generated and/or analyzed during the current study are not publicly available because the datasets contain multiple sensitive identifiers, but the datasets are available from the corresponding author on reasonable request.
