# Supplementary material for: Perinatal exposure to traffic related air pollutants and the risk of infection in the first six months of life: a cohort study from a low-middle income country
Source: Int Arch Occup Environ Health. 2024 Apr 17;97(5):575–86. doi: 10.1007/s00420-024-02064-0 (PMC11129992; doi:10.1007/s00420-024-02064-0)
Supplement: Supplementary file 1 — Supplementary file1 (DOCX 23 KB) [file 420_2024_2064_MOESM1_ESM.docx]

**Supplemental Table 1.** **LUR models for air pollutants concentration assessment**

| **Outdoor air pollutants** | **LUR models** |
| --- | --- |
| **PM_2.5_ (μg/m^3^)** | = 74.40 + (1.381 x 10^-4^ x t_motor) + (-2.63 x 10^-3^ x pop) + (4.769 x 10^-3^ x  t_heavyvehicle) |
| **Soot (10^-5^ per m)** | = 5.466 + (1.489 x 10^-5^ x t_motor) + (1.794 x 10^-3^ x total 500) + (- 2.591 x 10^-2^  x ISA) |
| **NO_x_ (μg/m^3^)** | = - 8,575 + (3.274 x 10^-4^ x t_motor) + (-7.842 x 10^-3^ x NDVI_1) + (80.78 x  y_c) + (3.441 x 10^-3^ x t_heavyvehicle) + (- 1.799 x 10^-4^ x pop) |
| **NO_2_ (μg/m^3^)** | = - 6,387 + (2.63 x 10^-4^ x t_motor) + (-6.132 x 10^-3^ x NDVI_1) + (60.22 x  y_c)+ (2.876 x 10^-3^ x min_500) + (- 1.478 x 10^-4^ x pop) |

**t_motor**: motor cycle density at the nearest street; **pop**: population density; **t_heavyvehicle**: heavy vehicle density such as trucks etc. at the nearest street; **t_ all** refer to systematic field observations (15 minute of traffic counting by a trained technician using a previously developed protocol). All other variables obtained from GIS databases. **Total 500**: major road length in a 500 m buffer (total refers to all open street map categories considered as major road, **ISA**: Impervious Surface Area; **NDVI**: normalized difference vegetation index , **y_c**: y coordinate of subjects’ house, **min_500**: road length of minor roads in a 500 m buffer.

**Supplemental Table 2. Correlation between air pollutants concentration at the measurement sites (n = 88)**

| **Air pollutant** | **PM_2.5_** | **PM_2.5_ absorbance** | **NOx** | **NO_2_** |
| --- | --- | --- | --- | --- |
| **PM_2.5_** | 1 | 0.51 | 0.03 | 0.14 |
| **Soot** | 0.51 | 1 | 0.16 | 0.14 |
| **NO_x_** | 0.03 | 0.16 | 1 | 0.87 |
| **NO_2_** | 0.03 | 0.14 | 0.87 | 1 |

all p values < 0.05

**Supplemental Table 3. Correlation between modelled air pollutants concentration exposure in**

**the cohort (n= 315)**

|  | **PM_2.5_** | **PM_2.5_ absorbance** | **NO_x_** | **NO_2_** |
| --- | --- | --- | --- | --- |
| **PM_2.5_** | 1 | 0.47** | 0.08** | 0.14* |
| **Soot** | 0.47** | 1 | 0.23** | 0.30** |
| **NO_x_** | 0.08** | 0.23** | 1 | 0.99** |
| **NO_2_** | 0.14* | 0.30** | 0.99** | 1 |

*= p< 0.05

** = p< 0.001

**Supplemental Table 4. The association between perinatal exposure to air pollutants with the incidence of all infections combined in the first six months of life (n= 298)**

| **Air pollutants concentration** |  | **Odd Ratio (95% Confidence interval)** | | | | |
| --- | --- | --- | --- | --- | --- | --- |
|  |  | **Infection at 1-2 months** | **Infection at 2-4 months** | | **Infection at 4-6 months** | **Cumulative infections 0-6 months** |
| **PM_2.5_** | Crude | 1.58 (0.96;2.62) | | 1.21 (0.78;1.87) | 1.47 (0.92;2.34) | 1.41 (0.78;2.54) |
|  | Adjusted | 1.34 (0.81;2.38) | | 1.22 (0.77;1.95) | 1.54 (0.93;2.53) | 1.53 (0.80;2.90) |
| **Soot** | Crude | 1.36 (0.93;1.99) | | 1.06 (0.77;1.46) | 1.33 (0.95;1.86) | 1.41 (0.92;2.17) |
|  | Adjusted | 1.43 (0.96;2.14) | | 1.00 (0.71:1.41) | 1.45 (1.00;2.17)* | 1.45 (0.90;2.34) |
| **NO_x_** | Crude | 0.71 (0.53;0.96) | | 1.00 (0.78;1.28) | 0.96 (0.75;1.24) | 0.82 (0.60;1.13) |
|  | Adjusted | 0.74 (0.54;1.07) | | 1.02 (0.79:1.33) | 0.99 (0.74;1.28) | 0.82 (0.58;1.15) |
| **NO_2_** | Crude | 0.73 (0.53;0.99) | | 1.01 (0.78;1.31) | 0.99 (0.76;1.29) | 0.83 (0.60;1.15) |
|  | Adjusted | 0.75 (0.54:1.03) | | 1.03 (0.78;1.35) | 1.00 (0.75:1.33) | 0.82 (0.58;1.17) |

All effect estimates of continuous data correspond to interquartile range (IQR) increase i.e. 7.14 μg/m^3^ for PM_2.5_, 0.74 x 10^-5^ per m for PM_2.5abs_, 4.47 μg/m^3^ for NO_x_, and 3.65 μg/m^3^ for NO_2._

**Adjusted to** mother’s age at pregnancy, socioeconomic status, mother working status during pregnancy, parity, delta BMI, active smoking, passive smoking, exposure to insecticides during pregnancy, gestational age, delivery complications, birth weight, breastfeeding.

*p= 0.05
